# Spawning salmon disrupt trophic coupling between wolves and ungulate prey in coastal British Columbia

**DOI:** 10.1186/1472-6785-8-14

**Published:** 2008-09-02

**Authors:** Chris T Darimont, Paul C Paquet, Thomas E Reimchen

**Affiliations:** 1 Department of Biology, Box 3020, Stn CSC, University of Victoria, Victoria, British Columbia, V8W 3N5, Canada,; 2 Raincoast Conservation Foundation, Box 77, Denny Island, British Columbia, V0T 1B0, Canada,; 3 Faculty of Environmental Design, Professional Faculties Building, Room 2182 University of Calgary, 2500 University Drive NW, Calgary, Alberta, T2N 1N4, Canada; 4 Department of Environmental Studies, 405 ISB, University of California, 1156 High Street, Santa Cruz, California, 95060, USA

## Abstract

**Background:**

As a cross-boundary resource subsidy, spawning salmon can strongly affect consumer and ecosystem ecology. Here we examine whether this marine resource can influence a terrestrial wolf-deer (*Canis lupus*-*Odocoileus hemionus*) predator-prey system in coastal British Columbia, Canada. Data on resource availability and resource use among eight wolf groups for three seasons over four years allow us to evaluate competing hypotheses that describe salmon as either an alternate resource, consumed in areas where deer are scarce, or as a targeted resource, consumed as a positive function of its availability. Faecal (n = 2203 wolf scats) and isotopic analyses (n = 60 wolf hair samples) provide independent data sets, also allowing us to examine how consistent these common techniques are in estimating foraging behaviour.

**Results:**

At the population level during spring and summer, deer remains occurred in roughly 90 and 95% of faeces respectively. When salmon become available in autumn, however, the population showed a pronounced dietary shift in which deer consumption among groups was negatively correlated (r = -0.77, P < 0.001) with consumption of salmon, which occurred in 40% of all faeces and up to 70% of faeces for some groups. This dietary shift as detected by faecal analysis was correlated with seasonal shifts in δ^13^C isotopic signatures (r = 0.78; P = 0.008), which were calculated by intra-hair comparisons between segments grown during summer and fall. The magnitude of this seasonal isotopic shift, our proxy for salmon use, was related primarily to estimates of salmon availability, not deer availability, among wolf groups.

**Conclusion:**

Concordance of faecal and isotopic data suggests our intra-hair isotopic methodology provides an accurate proxy for salmon consumption, and might reliably track seasonal dietary shifts in other consumer-resource systems. Use of salmon by wolves as a function of its abundance and the adaptive explanations we provide suggest a long-term and widespread association between wolves and salmon. Seasonally, this system departs from the common wolf-ungulate model. Broad ecological implications include the potential transmission of marine-based disease into terrestrial systems, the effects of marine subsidy on wolf-deer population dynamics, and the distribution of salmon nutrients by wolves into coastal ecosystems.

## Background

Subsidies of energy and nutrients across habitat boundaries can affect the behaviour and life history in a broad array of taxa [1-6]. The return of salmon (*Oncorhynchus *spp.) from oceanic environments to terrestrial spawning areas provides a striking example of such cross-boundary resource subsidy. Offering a predictable, nutritiously valuable, and spatially and temporally constrained food, salmon attract a diversity of terrestrial predators and scavengers [*e.g. *[7-17]].

Among multiple terrestrial users, only few capture salmon, transferring nutrients to adjacent shorelines and subsequent consumers. Although river otters (*Lontra canadensis*) and flooding activity contribute [9], critical to this process are bears (*Ursus *spp.), which partially consume salmon and deposit carcass remains as well as their urine and faeces (containing salmon-derived nutrients) throughout riparian areas [*e.g. *[7,11,18,19]]. This behaviour directly and indirectly provides nutrients to multiple trophic levels, including vegetation, through scavenging of carcasses followed by decay and subsequent fertilization of riparian vegetation [18,20-23]. If bears are the primary and most widely distributed vectors linking salmon to terrestrial environments, one might predict the ecological consequences based solely on these and similar studies of bear-salmon interactions.

Another terrestrial carnivore has been linked to salmon but their ecological relationship is not well understood. There are tangential observations of salmon as a food resource for wolves (*Canis lupus*), but none describing them as frequent prey [24-26]. In wolves of coastal and interior Alaska, however, Szepanski et al. [27] identified marine-enriched stable isotope signatures and suggested the dominant source was spawning salmon. With complementary results, Darimont and Reimchen [28] sampled chronologically segmented portions of guard hair from wolves across British Columbia (BC) and demonstrated that seasonal marine isotopic enrichment occurred during fall, when salmon became available. Subsequently, a survey of prey remains in wolf faeces across 60,000 km^2 ^of coastal BC detected the presence of salmon in about 7% of samples, even though sampling primarily occurred before the spawning season began [29]. Finally, behavioural evidence from coastal BC suggested that wolves are not simply scavengers but can efficiently prey on salmon [30]. Collectively, these observations suggest that wolves might also be a frequent and widespread predator of salmon and biological vector of salmon-derived nutrients.

Such a wolf-salmon association would depart from the dominant pattern defining this terrestrial carnivore. Recent reviews concluded that, although wolves are flexible and opportunistic predators, they primarily prey on ungulates – or hoofed animals – and ungulate presence and density in an area determines the distribution, behaviour, and ultimately reproduction and survival of wolves [31-33]. Consistent with this conclusion, Szepanski et al. [27] reasoned that the greater salmon consumption among wolves of mainland southeast Alaska they estimated was likely related to reduced ungulate (black-tailed deer; *Odocoileus hemionus*) availability; previous research had shown that deer densities were lower on the mainland compared with the islands. This supported the hypothesis that salmon were alternate prey to which wolves switch under conditions of low ungulate abundance. Likewise, it is consistent with broader theory that predators will switch to alternative prey when preferred foods are less available [34,35].

We address this hypothesis with resource use data from eight groups of wolves for three seasons over four years across a landscape that varies in availability of ungulates and salmon. We estimate resource use using two methods: i) identification of prey remains in wolf faeces and, ii) stable isotope analysis [*review in *[36]]. Relevant here, marine resources like salmon have higher carbon and nitrogen isotopic signatures compared with terrestrial foods, making a signal of marine resource use detectable in the tissues of consumers [37]. Primarily, we test whether wolves use salmon as a function of deer or salmon availability. Additionally, we examine how consistent faecal and isotopic data sets might be in estimating seasonal and intrapopulation variation in foraging. We show strong concordance between these data sets and that wolves target salmon as a function of salmon availability, not deer availability.

## Methods

### Study Area

**Figure 1:**
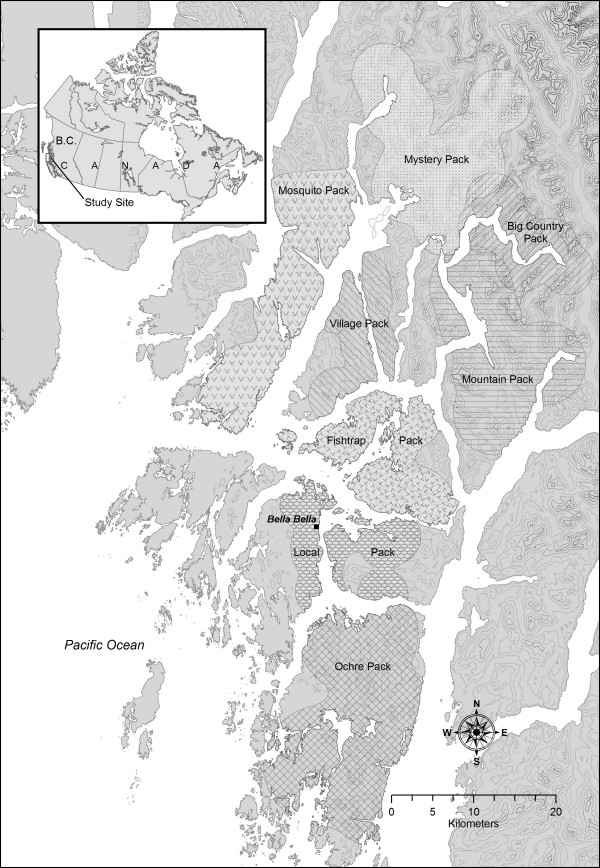
**Study area and home ranges of wolf social groups**. Study area in which wolves (*Canis lupus*) were sampled for hair and faeces on the central coast of British Columbia, 2001 to 2004. Home ranges estimated as 95% kernels based on re-sightings of individual wolves.

BC's central coast is a remote area, accessible only by boat or air, and only minimally modified by industrial activity [29]. Our study area is roughly 3,300 km^2^, and is centred on Bella Bella (52° 10' N, 128° 09' W; Figure 1).

### Assessing resource availability

To assess variation in resource availability among wolf groups, we first estimated home ranges using data on re-sightings of individuals. After hundreds of hours of direct observations that included videography and photography [*e.g. *[30,38]], differences among wolves in pelage and other morphological characters allowed us to identify repeatedly at least one member of each group over the 4 years of study. We used ArcView 3.2 to plot these re-sightings and used the 'Home Range' application to estimate 95% kernel home ranges [39]. This method might be limited by different probabilities of observing wolves among and within packs. Additionally, estimates cannot account for potential variation in home ranges among years. Nonetheless, we assume our results yield an adequate estimate of home range sizes and configurations to estimate relative resource availability for each pack (below). Indeed, microsatellite data extracted from wolf faeces collected in 2003 across five of these putative home ranges are yielding similar estimates for home range sizes and configurations (Erin Navid, University of Calgary, *unpublished data*).

To estimate deer availability, we applied a model [40,41] we previously developed that was based on the relationship between topographical slope and deer pellet density [42], derived from 110 km of transects conducted across our study area. Model output was converted to a spatial probability layer with which we calculated a relative deer density estimate (DEER), ranging from 0 to 1, for each home range.

To estimate salmon availability (SALMON), we extracted data from the Pacific Salmon Escapement Database (nuSEDS), maintained by Fisheries and Oceans Canada for each creek in each year. We then converted escapement numbers to biomass available in each 95% kernel home range using published weights for each species [43,44], and assuming a 1:1 sex ratio. The number of inventoried salmon creeks in each home range varied from 1 to 8. We assumed that wolves have equal access to each creek within their territories.

### Assessing resource use

During spring (May/early June), summer (late July), and fall (late September/early October), we collected wolf faeces on established transects. In 2001, we sampled the home ranges of four groups, and in 2002 and 2003, we added four more to total eight groups sampled each season and year. Within home ranges, sites were well-distributed and on average about half included creeks with spawning salmon.

During spring and summer 2001 to 2004, we also collected wolf hair that had been shed in resting beds on established transects or at 'homesites' (reproductive areas; [45]). Wolves have one annual moult that begins in late spring when the old coat sheds and a new one grows until late fall [24]. Therefore each hair sample's isotopic datum provides an integrated record of individual diet for roughly half the previous year. We assume each sample originated from one wolf, as they were collected from resting beds and on most occasions we sampled hair directly after viewing wolves.

Identification of prey remains used dichotomous keys [*e.g. *[46]] and followed protocols in Darimont et al. [30]. To eliminate inter-observer variability, only one person identified prey remains, and only after a lengthy training period (~60 hours). We estimated her precision by having an independent volunteer select 141 scats (~6%) for re-sampling, as well as administer and score the results. The primary prey item was consistently identified in 139 (98.6%).

Isotopic analysis of hair followed Darimont and Reimchen [28]. Isotopic signatures are expressed in delta notation (δ) as ratios relative to PeeDee limestone (carbon) and atmospheric N_2 _(nitrogen) standards as follows:

δX = [(R_sample_/R_standard_) - 1] * 1000,

where X is ^13^C or ^15^N, and R is the corresponding ratio ^13^C/^12^C or ^15^N/^14^N. Isotopic data are expressed in delta notation (δ) in ‰ units [36].

### Assessing resource use in context of resource availability

We used faecal data to document seasonal and intra-group differences in resource use but focus on isotopic data to test hypotheses of resource selection. For scat data, we report occurrence per faeces (O/F) for comparison with published literature but use occurrence per item (O/I) in statistical tests because the former can be problematic, as it exceeds unity when summed (because some faeces contain multiple items). O/F is the frequency item occurrence in all *faeces*, whereas O/I is the item's frequency among all *items *identified in all faeces. We also estimated mammalian biomass consumed using a regression equation created by Weaver [47]: Y = 0.439 + 0.008 X, where Y is the estimated biomass of prey consumed per faecal sample and X is the mass of prey. We used mean masses of adults [47-49], and assumed a 1:1 sex ratio. For deer, however, we distinguished between adults and fawns hair using diagnostic diameter and colour characters [50] and assigned fawn mass as 25% of adult mass. By necessity, biomass estimates excluded non-mammalian prey (n = 404 of 2692 items).

For isotopic data, we report signatures from whole hairs as well as in approximately equal distal and proximal segments (relative to root), which – given known moult chronology – provide proxies for summer and fall diets, respectively. We calculated any 'seasonal isotopic shifts' by subtracting summer from fall values [28]. In wolves that received δ^13^C and δ^15^N enrichment from salmon, which are available only during fall, one would expect positive seasonal isotopic shifts.

We used information theory to distinguish among competing hypotheses. Specifically, we developed a simple set of candidate models [weighted least squares general linear models (GLMs)] to examine how the availability of deer (DEER), salmon (SALMON), and their interaction might influence salmon use by wolves, and included year (YEAR) as a random term. We used the average δ^13^C seasonal isotopic shift of each group in each year as the dependent variable (n = 15 'group years') and proxy for salmon use for several reasons. First, faecal analyses might be sensitive to numerical and spatial sampling biases; faecal sample sizes varied considerably among 'pack seasons' (n = 9 to 132 scats) and might be biased to contain resources most available at the location of defaecation. In contrast, isotopic signatures incorporate many months of foraging behaviour. Second, we focused on δ^13^C because it is a much better tracer of dietary 'source' (*i.e. *marine versus terrestrial) than δ^15^N, which also reflects trophic position [50,51]. Third, if wolves used salmon, they should show elevated δ^13^C signatures in the fall-grown hair compared to summer-grown hair [28].

For each candidate model, we calculated Akaike Information Criteria (AIC), adjusted for small sample sizes, following the formula: AIC_c _= n log(*o*^2^) + 2K + 2K(K + 1)/(n - K - 1), where *o*^2 ^= Sum (e_i_^2^/n), K is the number of parameters (including intercept and error term), n the numbered of 'group years' and e_i _the residuals for each candidate model [[52], p. 63]. We then evaluated ΔAIC_c _to select best approximating model(s) and make appropriate inference, using ΔAIC_c _< 4 to describe the top model set. Finally, we summed Akaike weights (Σω_i_) across the top model set for each variable to rank them by importance [52]. δ^13^C seasonal isotopic shift data were normally distributed (Kolmogorov-Smirnov Z test; P = 0.35). Models were weighted by the square root of sample size for each 'group year'. Each candidate model had errors that were normally distributed (Kolmogorov-Smirnov Z tests, all P > 0.05). Tests were performed using SPSS 11.0 (SPSS Inc., Chicago, USA).

## Results

### Resource availability

Deer and salmon availability differed among groups. Average probabilities of detecting deer pellets across home ranges (our proxy for relative deer density) varied from 0.06 to 0.26 among the 8 social groups. More variation existed in salmon availability among 'group years', which ranged from approximately 1 to over 220 metric tonnes per group per year.

Resource availability can also be expressed in terms of nutrients in different foods groups. Although comparable in protein, salmon provide roughly 30% more fat than deer and more than four times the energetic content per unit mass (Table 1).

**Table 1:** Mean nutritional content in 100 grams of raw black-tailed deer and pink salmon.

**Content**	**Deer**	**Salmon**
Protein (g)	19.94	21.5
Fat (g)	2.66	3.45
Energy (kj)	111	485

Data from raw muscle tissue. Source: United States Department of Agriculture Nutrient Database .

### Resource use among seasons

**Figure 2:**
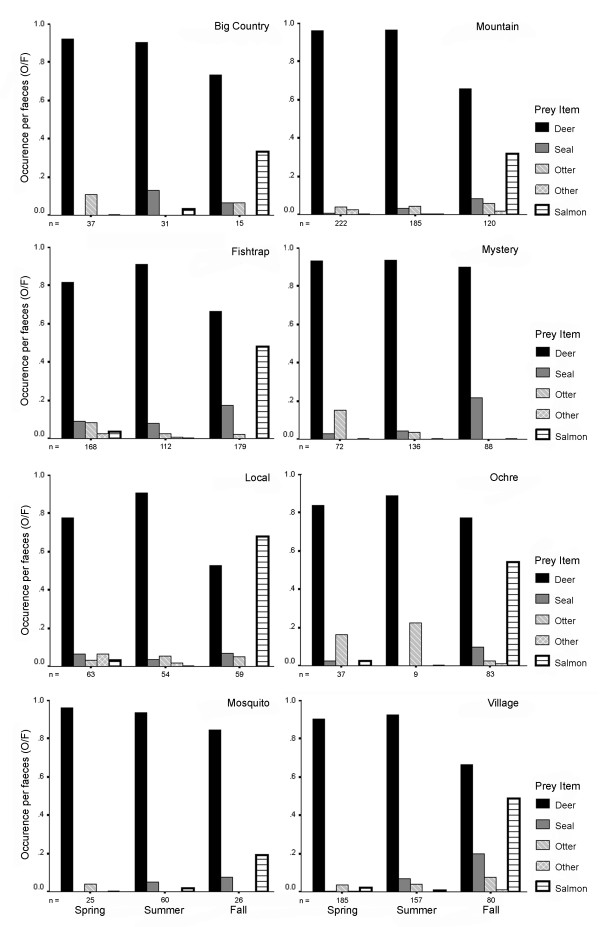
**Inter-group and -season variability in prey remains identified in wolf faeces**. Wolf (*Canis lupus*) faeces collected during spring, summer and fall, pooled across 2001 to 2003 in coastal British Columbia. Local, Ochre, Mosquito and Mystery groups sampled in 2002 and 2003 only. Remaining groups were sampled in all 3 years. 'Other' are prey as identified in Table 1. Occurrence per faeces measures the frequency of occurrence of an item among total faeces of each group.

**Figure 3:**
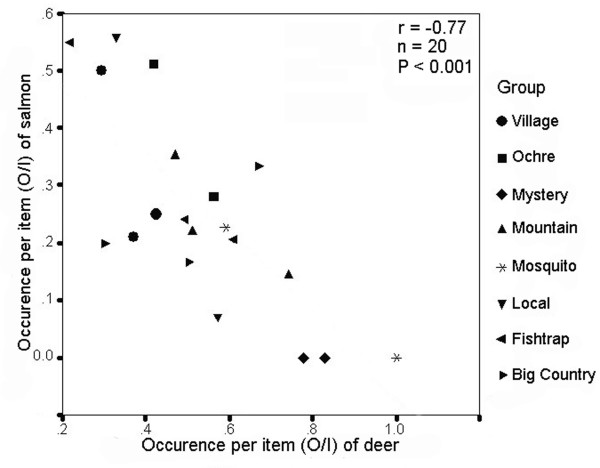
**Relationship between consumption of deer and salmon by wolves during autumn**. Occurrence per item (O/I) of deer (*Odocoileus hemionus*) and salmon (*Oncorhynchus *spp.) in wolf (*Canis lupus*) faeces collected during fall 2001 (n = 4 groups), 2002 (n = 8), and 2003 (n = 8) in coastal British Columbia. O/I measures the frequency of occurrence of an item among total items identified in a group's faeces during a given period (in this case, fall).

Faecal data (n = 2203 scats) collected over spring, summer, and fall showed strong seasonal patterns in resource use. Over all seasons combined and at the population level, resource use was broad but deer dominated diet, occurring in 90 to 95% of faeces during spring and summer (See additional file 1: Prey items identified in the faeces of wolves of coastal British Columbia). During fall, when salmon become available, however, the population diverged from a deer-dominated diet; for years pooled, population-level occurrence per item (O/I) of deer was significantly lower in the fall (ANOVA; F_2,21 _= 26.54, P < 0.001; Tamhane's T2 comparing fall with spring and summer, both P < 0.001). This difference was also significant in individual years (ANOVAs; all P < 0.005). Estimates of salmon occurrence per faeces (O/F) during fall averaged 40% and approached 70% for some groups (Figure 2; See additional file 1: Prey items identified in the faeces of wolves of coastal British Columbia). This pronounced shift in foraging behaviour to declining use of deer during fall was strongly related to salmon use; using 'group years' as cases, there was a strong inverse relationship between O/I of salmon and O/I of deer during fall (r = -0.77, n = 20, P < 0.001, Figure 3).

Isotopic data also showed seasonal variation in resource use, much of it related to salmon use. Whole hair δ^13^C values, indexing diet from the summer to fall, ranged from -24.4 to -16.8 (mean = -21.4, SD = 2.0), and δ^15^N ranged from 6.4 to 14.3 (mean = 9.5, SD = 2.1). Reflecting the marine nature of this variation, δ^13^C and δ^15^N were strongly correlated (r = 0.95, n = 60, P < 0.001).

**Figure 4:**
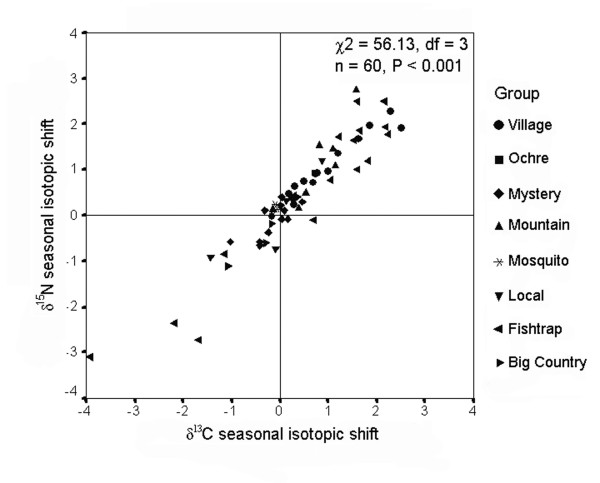
**Seasonal isotopic shifts in wolf hair**. Seasonal isotopic shifts in δ^13^C and δ^15^N in wolf (*Canis lupus*) hair, collected in coastal British Columbia, 2001 to 2004. Seasonal shifts calculated by subtracting values in distal (summer-grown) hair segments from basal (fall-grown) hair segments.

**Figure 5:**
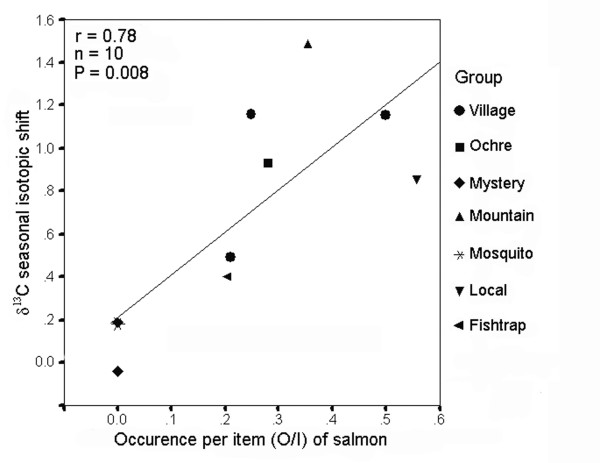
**Relationship between faecal and isotopic data to detect salmon use by wolves**. Salmon (*Oncorhynchus *spp.) remains in wolf (*Canis lupus*) faeces expressed as occurrence per item in each group during fall and the mean seasonal isotopic shift, which is the fall minus summer δ^13^C values in wolf hair, averaged among individuals of the same groups grown during the same year. Samples collected in coastal British Columbia, 2001 to 2004.

Three tests revealed that most marine-derived isotopic enrichment was incorporated during fall and associated with salmon. First, δ^13^C values in whole hair samples were correlated with seasonal (fall minus summer) isotopic shifts in δ^13^C in the same hair (r = 0.54, n = 15, P = 0.038). Second, most individuals showed positive seasonal isotopic shifts between summer and fall, occupy the region of isotopic niche space defined by greater δ^13^C and δ^15^N signatures during fall compared with summer (χ^2 ^= 56.13, df = 3, n = 60, P < 0.001; Figure 4). Third, we examined 'group years' with both faecal data during fall (n range: 9 to 92; mean = 54.0 faeces/group) and group-averaged δ^13^C seasonal isotopic shifts from wolf hair grown during that same year among members of those same groups (n range: 1 to 6; mean = 3.4 hair samples/group); cases with higher O/I salmon during fall showed greater average seasonal isotopic shifts in δ^13^C (r = 0.78, n = 10, P = 0.008, Figure 5).

### Inter-group variation in resource use

Groups varied in salmon use as assessed by both faecal and isotopic data. In a GLM, weighted by the square root of the number of items in all scats in each 'group season', variation among groups in O/I salmon during autumn approached significance (P = 0.051, Figure 2). In similar designs, but weighted by the square root of the number of hair samples used to compute averages for each 'group year', δ^13^C signatures in un-segmented wolf hair also differed among groups (P = 0.034), and approached significance for seasonal isotopic shifts in δ^13^C (P = 0.059).

### Resource use in context of resource availability

**Figure 6:**
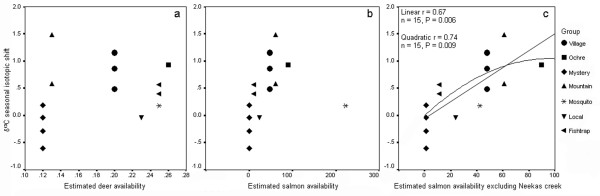
**Relationships between salmon use by wolves and the availability of deer and salmon**. Mean group δ ^13^C seasonal isotopic shift in wolf (*Canis lupus*) hair – a proxy for salmon use – as a function of estimated: a) deer (*Odocoileus hemionus*) availability, b) salmon (*Oncorhynchus *spp.) availability, and c) salmon availability in a data set in which the Mosquito group 2002 salmon estimate datum excluded the Neekas River, the most productive in the study area, but one where wolf sign was rarely observed during fall. Samples collected in coastal British Columbia, 2001 to 2004.

This variation in resource use among groups was relatively insensitive to estimated deer availability but correlated positively to salmon availability. In our first evaluation, using the entire dataset, SALMON and YEAR had the greatest utility in predicting the δ^13^C seasonal isotopic shift, our proxy for salmon use. Summing weights among top models ranked SALMON (Σω_i _= 0.61) marginally above YEAR (Σω_i _= 0.44), whereas DEER and DEER × SALMON were ranked much lower (both Σω_i _= 0.14). DEER occurred in only one top model, and with a positive parameter coefficient, suggesting – not consistent with either hypothesis – greater salmon use with greater deer availability. However, a bi-variate plot showed no linear relationship between DEER and δ^13^C seasonal isotopic shift (Figure 6a). Examination of parameter coefficients for SALMON revealed a strongly positive and significant effect, but only in the third model (See additional file 2: Top model sets to predict the use of salmon by wolves). A bi-variate plot between SALMON and δ^13^C seasonal isotopic shift showed how an outlier SALMON datum (Mosquito group 2002) influenced results (Figure 6b).

We re-evaluated candidate models but modified the Mosquito 2002 outlier datum to exclude the contribution of the Neekas watershed to the home range's SALMON estimate. During years studied, the Neekas yielded an average of 179.9 tonnes of salmon per year, representing 81% of biomass in their home range and alone doubled the total biomass available to any group. Yet, Mosquito wolves infrequently used this watershed; the proportion of Mosquito group's faeces collected there during autumn was very low; we collected only 2 faeces in 2003 and none in 2002. With this modification, our analysis revealed that SALMON was clearly the best predictor of salmon use. The preferred model (lowest ΔAIC_c_) included only SALMON (and the intercept; ω_i _= 0.57, See additional file 2: Top model sets to predict the use of salmon by wolves). SALMON (Σω_i _= 0.75) outranked YEAR (Σω_i _= 0.23) and DEER (Σω_i _= 0.09) by factors of about 3.3 and 8.3 respectively. A bi-variate plot (Figure 6c) revealed a significant and positive correlation between δ^13^C seasonal isotopic shift and SALMON to which a linear (r = 0.67, n = 15, P = 0.006) and quadratic form (r = 0.74, n = 15, P = 0.009) could be fit.

## Discussion

Determining which resources are used in the context of their availability provides fundamental life history information and can yield insight into the ecological relationships among consumer, prey, and the ecosystem. Consistent with prevailing knowledge about wolf-prey systems, for much of the year wolves of coastal BC are closely tied to ungulate prey. During autumn, however, an alternate predator-prey system emerged with previously undocumented ecological detail. When salmon became available seasonally, we observed a population-level shift in resource use as indicated by two independent datasets. Associations between the occurrence of salmon in fall faeces and seasonal isotopic shifts were significant and moderately strong. This suggests the intra-hair methodology [28] offers an accurate proxy for salmon consumption, and perhaps also for tracking seasonal dietary shifts in other predator-prey systems.

Many systems receive pulsed food resources, which decay in abundance over time. Because there are long durations between pulses, theory predicts that few consumers will be specialists on such resources. Instead, generalist consumers should be most likely to respond [53]. Across their remaining Holarctic distribution, although wolves are opportunistic and able to subsist on alternate foods such as beaver, livestock or even garbage, close ecological and evolutionary associations with ungulate prey are the norm [31-33]. With this perspective, it follows that any departure from a diet dominated by ungulates might occur only during times or in areas of low ungulate availability.

In contrast, our data suggest salmon are a targeted resource. Salmon availability clearly outperformed deer availability in predicting use of salmon. Although not a highly important variable, there was variation in salmon use among years. This could represent many conditions that might change yearly, including climate (and deer vulnerability) and competitive interactions (below).

How we estimated resource availability influences interpretation of results. Manly et al. [54] cautioned researchers to carefully consider difference between resource *availability *and *abundance*. Our deer model yielded a coarse estimate of relative deer abundance across large home ranges, and one that does not vary among years. Actual availability (*i.e. *numbers and vulnerability) might be different. For example, coastal black-tailed deer have phenotypes that are resident at low elevations year round and those that seasonally migrate to higher elevations during summer [55]. Differences among home ranges in the proportion of these phenotypes might influence the availability of deer to wolves. Regardless, the positive correlation between salmon availability and use is straightforward, and alone provides support to differentiate between hypotheses.

### Adaptive explanations for use of salmon

Whereas this wolf-prey association during fall departs from a 'wolf-ungulate' model, it is consistent with adaptive explanations based on safety, nutrition, and energetics. Selecting benign prey such as salmon over potentially dangerous ungulate prey follows predictions of foraging theory [56]. While hunting ungulates, wolves commonly incur serious and often fatal injuries [31].

In addition to safety benefits, we show here that salmon also provides enhanced nutrition over deer, especially in fat and energy. Moreover, strict comparisons might underestimate the nutritional value of salmon. Wolves selectively consume lipid-rich heads [30] and potentially benefit from docosahexaenoic acid, an omega-3 fatty acid, which is critical for nervous system function, can be manufactured only from dietary sources, and occurs at high levels in brain and optic tissue [57]. Finally, for equivalent energetic intake, wolves face less handling time and need to travel far less for salmon compared with searching for vulnerable ungulate prey [*e.g. *[58]]. If we consider energetic content as a central currency, and given a ratio of its value per mass of pink salmon compared with deer (4.4:1, calculated from Table 1) and an estimated daily requirement of 2.7 kilograms of deer per wolf of average mass per day among coastal populations [59], wolves that forgo deer would on average require only 0.62 kg of pink salmon each day. If wolves consume exclusively salmon heads that comprise (a conservatively estimated) 10% of the average mass of pink salmon in the area [1.3 kg; [43,44]], these energetic requirements would be satisfied by capturing only 4.6 salmon per day.

### Processes that might constrain use of salmon

These safety, nutritional, and energetic benefits conferred in a spatially-constrained food resource would promote competition with other salmon consumers. Brown and black bears have been observed in several competitive interactions with wolves over resources [*e.g. *[60]], including salmon [61]. Such interactions might be most intense under conditions of high resource density, and could explain why wolves avoid the Neekas River, which hosts extraordinarily high salmon density (in fact the highest on the entire BC coast [biomass/km]). Likewise, such competitive interactions across the study area might also explain the decline in slope in seasonal isotopic shift at higher salmon abundances (*i.e. *fit to a quadratic form).

Additional processes might also limit the use of salmon by wolves. First, wolves might be compelled to partition their diet, perhaps requiring a particular suite of micronutrients in deer or avoiding the accumulation of others in salmon. Disease, specifically 'salmon-poisoning disease' (*Neorickettsia helminthoeca*), which in high quantities is fatal to canids, might also play a role [[30]*and references therein*]. Third, focusing on a spatially-constrained resource might create opportunity costs of not patrolling and defending larger portions of their territories.

### Ecological implications

Based on relationships we show between availability and use, we predict salmon consumption is widespread wherever wolves and salmon still exist [*see also *[29,30]]. Accordingly, we expect higher-order ecological implications, similar to those initiated by wolves in other systems. For example, by preying on large ungulates, wolves indirectly provide a considerable proportion of carcasses to a diversity of scavengers, including coyotes (*C. latrans*), bears, and ravens (*Corvus corax*) [62,63]. Notable differences, however, exist between unused portions of ungulate and salmon carcasses. First, remains of salmon are not defended by wolves [30], and thus the carrion is immediately available. Second, because carcasses are relatively small and can be more readily dispersed, more individual (vertebrate) scavengers likely gain access to salmon compared with large (ungulate) carcasses over which multiple scavengers might compete. As a consequence, this subsidy might be more evenly and broadly dispersed. Finally, the resource subsidy offered by this terrestrial carnivore is one transported across a boundary of land and sea.

This wolf-provided subsidy of salmon to terrestrial ecosystems also differs from that provided by bear vectors. In contrast to wolves, which often forage among or near family members, carcass transport by bears is thought to be mediated by intra-specific competition. As a consequence, one might expect different spatial patterns of nutrient subsidy. In a black bear system, Reimchen [18] observed that about 80% of salmon were transferred up to 100 m into the forest, with larger and fresher male carcasses transported further. In contrast, in 70% of previously observed transport events by wolves, carcasses were deposited on estuarine grasses, within a few metres of the creek [30]. Moreover, tissue content in abandoned carcasses also differs. Whereas wolves target head tissue, bears target brains and eggs, and under conditions of relatively low salmon abundance also consume musculature [18]. Consequently, on average more tissue (of greater energetic content) would be available to scavengers of wolf-provided carcasses.

The most notable difference between wolves and bears is the distribution of these vectors across the landscape of coastal BC. Brown bears occur on the mainland, and in low densities and frequencies on inner islands; black bears commonly inhabit mainland and inner islands, but are largely absent on outer islands [64]. In contrast, wolves occur on all landmasses [40]. Therefore, wolves might be the primary biological vector on some islands, particularly isolated outer islands. Given the behavioural differences among vectors, this distributional pattern would increase and alter the 'resource shed' into which salmon are transported by terrestrial vectors [65].

Wolf-salmon associations might have additional ecological implications, namely in disease ecology and terrestrial predator-prey dynamics. In addition to *N. helminthoeca*, our pilot work on diseases [H. Bryan, University of Saskatoon, *unpublished data*] has shown that wolves in areas and periods of greater salmon consumption have higher prevalence of eggs from *Dyphyllobothrium *spp. This fish tapeworm uses piscivorous terrestrial mammals as final hosts in its life-cycle, which crosses the marine-terrestrial boundary. Additionally, we suspect that wolves subsidized by marine prey such as salmon might limit deer populations [29]. Under allochthonous resource supply, densities (and ecological influence) of consumers can be greater than predicted by *in situ *productivity [1]. This hypothesis would be especially plausible on islands where deer productivity and/or immigration from other landmasses might not offset predation [66].

## Conclusion

Our data suggest that salmon are a targeted resource in our study area and likely wherever wolves and salmon still co-occur. This coupled with the adaptive explanations we present argue for an historical predator-prey association with broad ecological implications. The future and nature of this (formally geographically widespread) wolf-salmon association is uncertain, however, given multiple threats posed to salmon systems. These include overexploitation by fisheries and destruction of spawning habitat [67], as well as diseases from exotic salmon aquaculture [68] that collectively have lead to coast-wide declines up to 90% over the last century [69].

## Authors' contributions

CTD lead fieldwork, conducted statistical analyses, and drafted the manuscript. All authors participated in study design and manuscript revision, and approved the final manuscript. This work was part of the Salmon Forest Project, conceived by TER.

## Supplementary Material

Additional file 1 Prey items identified in the faeces of wolves of coastal British Columbia. Click here for file

Additional file 2 Top model sets to predict the use of salmon by wolves. Click here for file
