# Supplementary material for: Spawning salmon disrupt trophic coupling between wolves and ungulate prey in coastal British Columbia
Source: BMC Ecol. 2008 Sep 2;8:14. doi: 10.1186/1472-6785-8-14 (PMC2542989; doi:10.1186/1472-6785-8-14)
Supplement: Additional file 1 — Prey items identified in the faeces of wolves of coastal British Columbia. [file 1472-6785-8-14-S1.doc]

## Additional File 1. Prey items identified in the faeces of wolves of coastal British Columbia.

|  |  | **Spring**  (n = 809, 891) | | | |  |  | **Summer**  (n = 744, 817) | | |  |  | **Fall**  (n = 650, 984) | | |  | |  | **Total**  (n = 2203, 2692) | | |
| --- | --- | --- | --- | --- | --- | --- | --- | --- | --- | --- | --- | --- | --- | --- | --- | --- | --- | --- | --- | --- | --- |
| **Prey** | | | **O/F**    **(%)** | **O/I**  **(%)** | **Biomass**  **(%)** | **O/F**  **(%)** | | | **O/I**  **(%)** | **Biomass**  **(%)** | | **O/F**  **(%)** | | **O/I**  **(%)** | **Biomass**  **(%)** | | **O/F**  **(%)** | | | **O/I**  **(%)** | **Biomass**  **(%)** |
| ***Terrestrial*** | | |  |  |  |  | | |  |  | |  | |  |  | |  | | |  |  |
| Deer | | | 82.1 | 74.5 | 83.6 | 44.4 | | | 40.4 | 52.9 | | 65.8 | | 43.5 | 75.7 | | 64.5 | | | 52.8 | 71.7 |
| Fawn | | | 7.2 | 6.5 | 4.5 | 49.1 | | | 44.7 | 35.6 | | 4.5 | | 3.0 | 3.1 | | 20.5 | | | 16.8 | 13.9 |
| Black Bear | | | 1.9 | 1.7 | 2.9 | 1.7 | | | 1.6 | 3.1 | | 1.1 | | 0.7 | 2.0 | | 1.6 | | | 1.3 | 2.7 |
| Bird | | | 1.0 | 0.9 | N/A | 0.7 | | | 0.6 | N/A | | 2.2 | | 1.4 | N/A | | 1.2 | | | 1.0 | N/A |
| Marten | | | 0.9 | 0.8 | 0.4 | 0.7 | | | 0.6 | 0.1 | | 2.0 | | 1.3 | 0.9 | | 1.1 | | | 0.9 | 0.4 |
| Beaver | | | 0.9 | 0.8 | 0.5 | 0.5 | | | 0.5 | 0.3 | | 0.8 | | 0.5 | 0.5 | | 0.7 | | | 0.6 | 0.4 |
| Rodent | | | <0.1 | <0.1 | <0.1 | 0.1 | | | 0.1 | <0.1 | | 0.3 | | 0.2 | 0.1 | | 0.1 | | | 0.1 | <0.1 |
| Goat | | | <0.1 | <0.1 | <0.1 | <0.1 | | | <0.1 | <0.1 | | 0.2 | | 0.1 | 0.2 | | <0.1 | | | <0.1 | <0.1 |
| Vegetation | | | 0.1 | 0.1 | N/A | 1.9 | | | 1.7 | N/A | | 6.9 | | 4.8 | N/A | | 2.7 | | | 2.2 | N/A |
| *Sub-total* | | |  | ***85.3*** | ***91.8*** |  | | | ***90.2*** | ***92.1*** | |  | | ***55.5*** | ***82.5*** | |  | | | ***75.7*** | ***89.3*** |
|  | | |  |  |  |  | | |  |  | |  | |  |  | |  | | |  |  |
| ***Marine*** | | |  |  |  |  | | |  |  | |  | |  |  | |  | | |  |  |
| Salmon | | | 1.6 | 1.5 | N/A | 0.4 | | | 0.4 | N/A | | 39.7 | | 26.2 | N/A | | 12.4 | | | 10.2 | N/A |
| Seal | | | 3.1 | 2.8 | 2.6 | 5.5 | | | 5.0 | 5.6 | | 14 | | 9.3 | 14.1 | | 7.1 | | | 5.8 | 6.8 |
| Otter | | | 6.4 | 5.8 | 3.0 | 3.4 | | | 3.1 | 1.7 | | 3.4 | | 2.2 | 1.9 | | 4.5 | | | 3.7 | 2.3 |
| Inter-tidal | | | 0.9 | 0.8 | N/A | 0.5 | | | 0.5 | N/A | | 4.6 | | 3.1 | N/A | | 1.9 | | | 1.5 | N/A |
| Sea Lion | | | 0.9 | 0.8 | 2.6 | 0.1 | | | 0.1 | 0.6 | | 0.6 | | 0.4 | 1.5 | | 0.5 | | | 0.4 | 1.7 |
| *Sub-total* | | |  | ***11.7*** | ***8.2*** |  | | | ***9.1*** | ***7.9*** | |  | | ***41.2*** | ***17.5*** | |  | | | ***21.6*** | ***10.7*** |
|  | | |  |  |  |  | | |  |  | |  | |  |  | |  | | |  |  |
| Other | | | 3.3 | 2.7 | N/A | 0.8 | | | 0.7 | N/A | | 5.4 | | 3.6 | N/A | | 3.1 | | | 2.5 | N/A |
|  | | |  |  |  |  | | |  |  | |  | |  |  | |  | | |  |  |

Scat samples (n = 2203) in spring, summer, and fall of 2001 to 2003. O/F is the occurrence per faeces and O/I is occurrence per item among the total items in all faeces. Percent biomass applied only to mammalian prey remains. ‘Other’ includes unidentified or rare items (*e.g*. beetles, Coleoptera). Items arranged in descending order of O/F and O/I for all seasons combined. n = # of faeces, # of items.
