# Supplementary material for: Spawning salmon disrupt trophic coupling between wolves and ungulate prey in coastal British Columbia
Source: BMC Ecol. 2008 Sep 2;8:14. doi: 10.1186/1472-6785-8-14 (PMC2542989; doi:10.1186/1472-6785-8-14)
Supplement: Additional file 2 — Top model sets to predict the use of salmon by wolves. [file 1472-6785-8-14-S2.doc]

## Additional File 2. Top model sets to predict the use of salmon by wolves

| **Data Set** | **Model Form** | **ΔAIC_c_** | **ω_i_** | **β_0_** | **S.E.** | **SALMON** | **S.E.** | **DEER** | **S.E.** | **SALMON**  **x DEER** | **S.E** | **R^2^** |
| --- | --- | --- | --- | --- | --- | --- | --- | --- | --- | --- | --- | --- |
| All data | β_0_ + (SALMON) | 0.000 | 0.41 | 0.452** | 0.177 | 0.002 | 0.003 |  |  |  |  | 0.43 |
|  | β_0_ + (YEAR) | 0.129 | 0.38 | 0.494 | 0.435 |  |  |  |  |  |  | 0.40 |
|  | β_0_ + (SALMON) + (DEER) + (SALMON x DEER) | 2.101 | 0.14 | -0.787 | 0.428 | 0.048** | 0.011 | 5.574* | 2.141 | -0.197** | 0.047 | 0.64 |
|  | β_0_ + (SALMON) + (YEAR) | 3.808 | 0.06 | 0.429 | 0.473 | 0.001 | 0.003 |  |  |  |  | 0.57 |
|  |  |  |  |  |  |  |  |  |  |  |  |  |
| Neekas River Salmon biomass excluded from 2002 Mosquito group datum | β_0_ + (SALMON) | 0.000 | 0.57 | 0.121 | 0.168 | 0.014** | 0.004 |  |  |  |  | 0.43 |
|  | β_0_ + (SALMON) + (YEAR) | 2.824 | 0.14 | -0.270 | 0.399 | 0.017** | 0.005 |  |  |  |  | 0.54 |
|  | β_0_ + (YEAR) | 3.622 | 0.09 | 0.494 | 0.435 |  |  |  |  |  |  | 0.14 |
|  | β_0_ + (DEER) + (SALMON) | 3.742 | 0.09 | -0.028 | 0.434 | 0.014* | 0.005 | 0.835 | 2.226 |  |  | 0.44 |

Wolves (*Canis lupus*) sampled from eight social groups, 2000 to 2003, in coastal British Columbia. Shown are model structure, ∆AIC_c_, and Akaike weight (ω_i_). SALMON is salmon biomass estimate in metric tonnes, and DEER is relative abundance of deer available to each pack, estimated from a model derived from deer pellet group data in study area. YEAR is a random factor. Shown also are parameter coefficients (for SALMON and DEER), their standard errors, and model R^2^. **p < 0.01, *p < 0.05. Shown are results using all data, and a dataset with the 2002 Mosquito group datum modified to exclude the contribution of salmon biomass from the Neekas River, where wolves infrequently occured.
